# Restriction Endonucleases from Invasive *Neisseria gonorrhoeae* Cause Double-Strand Breaks and Distort Mitosis in Epithelial Cells during Infection

**DOI:** 10.1371/journal.pone.0114208

**Published:** 2014-12-02

**Authors:** Linda Weyler, Mattias Engelbrecht, Manuel Mata Forsberg, Karl Brehwens, Daniel Vare, Katarina Vielfort, Andrzej Wojcik, Helena Aro

**Affiliations:** 1 Department of Molecular Biosciences, The Wenner-Gren Institute, Stockholm University, 106 91 Stockholm, Sweden; 2 Department of Chemistry and Molecular Biology, University of Gothenburg, 405 30 Gothenburg, Sweden; University of Würzburg, Germany

## Abstract

The host epithelium is both a barrier against, and the target for microbial infections. Maintaining regulated cell growth ensures an intact protective layer towards microbial-induced cellular damage. *Neisseria gonorrhoeae* infections disrupt host cell cycle regulation machinery and the infection causes DNA double strand breaks that delay progression through the G2/M phase. We show that intracellular gonococci upregulate and release restriction endonucleases that enter the nucleus and damage human chromosomal DNA. Bacterial lysates containing restriction endonucleases were able to fragment genomic DNA as detected by PFGE. Lysates were also microinjected into the cytoplasm of cells in interphase and after 20 h, DNA double strand breaks were identified by 53BP1 staining. In addition, by using live-cell microscopy and NHS-ester stained live gonococci we visualized the subcellular location of the bacteria upon mitosis. Infected cells show dysregulation of the spindle assembly checkpoint proteins MAD1 and MAD2, impaired and prolonged M-phase, nuclear swelling, micronuclei formation and chromosomal instability. These data highlight basic molecular functions of how gonococcal infections affect host cell cycle regulation, cause DNA double strand breaks and predispose cellular malignancies.

## Introduction

*Neisseria gonorrhoeae*, the causative agent of the sexually transmitted disease gonorrhea, efficiently adheres to and invades epithelial cells of the urogenital tract. The initial bacterial adherence is dependent on pilus expression that is followed by a multitude of secondary receptors. High antigenic variations, low-immune response, survival inside neutrophils and anti-apoptotic features among certain strains ensure increased survival and persistence [1], [2]. Approximately half of infected women and 10% of the men are asymptomatic carriers of the disease, leading to long-term infections, delayed initiation of antibiotic treatment, and secondary complications such as prostatitis, pelvic inflammatory diseases, ectopic pregnancies, and sterility.

Repeating- and long-term gonococcal infections have been associated urogenital cancers, such as cervical, prostate, or anal cancer [3]–[8], although the molecular mechanisms behind this are largely unknown. In order to dissect the molecular mechanisms of which gonococcal infection may cause cellular malignancies, we have previously shown that an infection results in cells deprived in adequate levels of cyclins [9]. The gonococcal infection causes also damage to the human genome by 700 DNA strand breaks per cell and hour in VK2/E6E7 epithelial cells, levels comparable to an acute dose of 1Gy of γ-irradiation. DNA double strand breaks (DSBs) were detected by increased level of 53BP1 foci that co-localized with γ-H2AX and were accompanied by downregulation of p53 [10]. *N. gonorrhoeae* infection also leads to upregulation and alternative processing of the human growth factor amphiregulin, a protein that is frequently upregulated in various types of cancer [11].

The host epithelium is a barrier against microbial infections. Maintaining regulated cell growth ensures an intact protective layer towards microbial-induced cellular damage. The cell cycle progression is tightly regulated by cyclins and their cognate cyclin dependent kinases (CDKs) and checkpoint proteins, such as the cyclin dependent kinase inhibitors (CKIs) p21 and p27, ensures the proper cellular growth and division [12], [13]. The mitosis is regulated by the anaphase promoting complex/cyclosome (APC/C) that, together with CDC20, interacts with mitotic checkpoint proteins such as MAD 1 mitotic arrest deficient-like 1 and MAD2 mitotic arrest deficient-like 1 to ensure no premature sister chromatid separation. Despite the rigorous control of cell cycle and division, many pathogenic bacteria produce cyclomodulins [14] to disturb the host cell cycle and also nucleomodulins to subvert host defenses by interfering with transcription, chromatin remodeling, RNA splicing and DNA replication and repair [15].

Here, we investigated the mechanism by which *N. gonorrhoeae* damages host DNA and what consequences this DNA damage had on mitosis. We present data showing that the restriction endonucleases produced by the invasive bacteria are upregulated and released during infection and transverse through the nuclear pores to reach and damage host cellular DNA. As a consequence, infected cells show impaired and prolonged M-phase due distortion of the spindle assembly checkpoint proteins MAD1 and MAD2, nuclear swelling, micronuclei formations, and lagging chromosomes.

Over all, the rapid increase in new cases and the emerged prevalence in antibiotic resistance strains will in near future most likely result in an population with long-term non-treatable infections and secondary complications. Hence, a greater understanding in the molecular functions behind the association between gonococcal infections and malignancies need to be prioritized. Therefore is of great importance to monitor *N. gonorrhoeae* to elucidate its potential role in carcinogenesis.

## Materials and Methods

### Cell lines and growth conditions

The immortalized human vaginal epithelial cell line VK2/E6E7 (ATCC CRL-2616, LGC Standards, London) has been derived from normal vaginal mucosal tissue and shows characteristics of stratified squamous non-keratinizing epithelia. The cell line was previously shown to be a good model for gonococcal adhesion [10], [16]. VK2/E6E7 cells were cultured in keratinocyte-serum free medium (K-SFM) supplemented with 0.1 ng/ml of human recombinant epidermal growth factor (Invitrogen, Carlsbad, CA, USA), 0.05 mg/ml of bovine pituitary extract (Invitrogen, Carlsbad, CA, USA), and 44.1 mg/l of calcium chloride (Sigma-Aldrich Inc., St. Louis, MO, USA). Cells were maintained at 37°C in 5% CO_2_. In all assays, monolayers of 30–40% confluency cells were used to avoid disturbing the progression of the cell cycle.

### Bacterial strain and infection assay

Piliated (P+) *Neisseria gonorrhoeae* strain MS11mk [17] is DNAse negative and referred to in the literature as MS11 P+. Bacteria were grown at 37°C in 5% CO_2_ on gonococcal medium base (GCB; Neogen, Lansing, MI, USA) agar plates containing Kellogg's supplement [18]. Piliated, non-opaque phenotypes were distinguished by morphology under a binocular light microscope. The choice to use non-opaque and piliated bacteria during infection was based on previous observations that (i) Opa expression in opaque cells increases bacterium-bacterium interactions to form larger microcolonies, thereby reducing bacterial uptake efficiency in vitro; and that (ii) pili play a major role not only in adhesion but also in invasion [19] [20]. For infection assays, 18- to 22-h-old piliated bacteria were collected from GCB agar plates and resuspended in PBS. Optical densities were measured at 600 nm to calculate the number of bacteria/ml. A multiplicity of infection (MOI) of 50 bacteria/cell was used in all experiments, unless stated otherwise. Bacteria were added to subconfluent monolayers of VK2/E6E7 cells and grown 37°C in 5% CO_2_ for 2–24 h. Subsequently, cell monolayers were extensively washed with PBS until no unbound bacteria were visible by light microscopy. Infected cells were then transferred for further analysis described below.

### Quantitative real time PCR analysis of bacterial gene expression

VK2/E6E7 cells were infected with bacteria for 2, 7, or 24 h. A MOI of 100 was used to ensure high quantities of bacterial RNA for qPCR analysis. As a negative control, the same numbers of bacteria were grown in K-SFM medium without VK2/E6E7 cells for the same time points. RNA was stabilized using RNAprotect Bacteria Reagent, and bacteria were lysed according to the manufacturer's recommendations. Bacterial RNA was isolated with the RNeasy Mini kit and total RNA (up to 1 µg) was reversed transcribed using the Transcriptor First Strand cDNA Synthesis Kit and random hexamer primers (all reagents from Qiagen). Primers for amplification of restriction endonucleases from *N. gonorrhoeae* MS11 were designed using genome sequences published by the European Nucleotide Archive [21], and primer sequences are listed in Table 1. PCR amplification was performed using a Light Cycler 480 and SYBR Green I Master Kit with 0.1 µM of each gene-specific primer and 5 µl of cDNA. Cycling conditions for PCR were as follows: initial denaturation for 5 min, followed by 30 cycles of denaturation at 94°C for 30 s, annealing at 55°C for 15 s, and extension at 71°C for 30 s. The ramp rate was 2.2–4.4°C/s. Relative expression levels of target gene mRNAs were calculated, normalized, and compared between MS11P+ alone and MS11P+ from infected cells. Elongation factor thermo unstable 1 (*Ef-Tu1*) and phosphoglucomutase (*pgm*) were used as standards for normalizing the relative expression of target genes.

**Table 1:** Primer sequences used for detection of bacterial genes by qPCR.

Gene	Primer orientation	Primer sequence
*NgoPII*	Forward	5′- CGG GGA TGC CAT CGA AGT TAA GAA -3′
	Reverse	5′- CCG CAT CTT TGC AGG CTT TTG T -3′
*NgoI*	Forward	5′- AAA CGC TGG AGG GAT GAA ATC TGC -3′
	Reverse	5′- AAA CGC TGG AGG GAT GAA ATC TGC -3′
*NgoNlaIV*	Forward	5′- ACC CAA ATG CCT CCC GAT TTC T -3′
	Reverse	5′- TAT CAA AAC CCG GGC AGG CAT T -3′
*NgoMIV*	Forward	5′- TTG CGT GCG GGT AAT GGC AAT A -3′
	Reverse	5′- TCC TTC AGA ACG GGC ATT TTG AGC -3′
*Ef-Tu1*	Forward	5′- TAC CGT CCC CAA TTC TAC TTC CGT-3′
	Reverse	5′- TTC CAT AGC GAT AGG CGC AAT CAG-3′

### Plasmid digestion

Bacterial colonies were harvested from GCB plates and resuspended in PBS to OD_600_ = 1. Bacteria were lysed by subjecting them to freeze-thaw cycles, which was confirmed by spreading 100 µl of lysates on GCB plates and incubating them overnight at 37°C in 5% CO_2_. Portions of lysates were further heat inactivated (HI) at 95°C for 10 min. One µg of the commercial plasmid pECFP-N1 (Clonetech, CA, USA) was subjected to either MS11 P+ lysate or HI lysate together with CutSmart buffer (New England Biolabs, Ipswich, MA, USA) for 1 h. As controls, circular/uncut pECFP-N1 was used as well as HindIII (Roche, Mannheim, Germany) linearized pECFP-N1. The plasmid reactions were run on 1% agarose gel electrophoresis in 1xTBE buffer and stained with ethidium bromide.

### Pulsed-field gel electrophoresis

Genomic DNA from VK2/E6E7 cells was extracted using the Wizard Genomic DNA Purification Kit (Promega, Madison, WI, USA), according to the manufacturer's recommendations. Extracted genomic DNAs were 100–250 kb in length. Bacterial colonies were harvested from GCB plates, resuspended in PBS to OD_600_ = 1, and lysed by subjecting them to freeze-thaw cycles. Portions of lysates were further heat inactivated (HI) at 95°C for 10 min. For each reaction, 1 µg of VK2/E6E7 DNA (equivalent to 230,000 cells) was incubated with 45 µl bacterial lysates in a final volume of 50 µl for 24 h at 37°C and 5% CO_2_. As positive controls, 0.5 µl each of the restriction enzymes NgoMIV, BamHI, KpnI and MfeI (New England Biolabs, Ipswich, MA, USA) and 5 µl CutSmart buffer were added to 1 µg genomic DNA in 50 µl reactions. Following digestion, 50 µl of In-Cert low melting agarose (2%) was added to each reaction, and 90 µl of the resultant mixtures were placed into agarose plugs. Inserts were loaded onto a Megabase agarose separation gel (1% chromosomal agarose, Bio-Rad). DNA was separated (120° field angle, 240 s switch time, 4 V/cm, 14°C) for 20 h. Gels was stained with ethidium bromide overnight at 4°C and subsequently examined in a UV illuminator. DNA fragmentation was measured with a Dynamic ROI profiler plug-in (ImageJ, NIH, USA).

### Microinjection and 53BP1 staining

Bacterial colonies were harvested from GCB plates and resuspended in PBS to OD_600_ = 1. Bacteria were subjected to repeated freeze-thaw cycles in order to accomplish lysis. Prior to microinjection, lysates were mixed with FITC-coupled Dextran (0.5 mg/ml), centrifuged for 15,000×g for 10 min, and loaded into Femtotips II injection needles (Eppendorf, Hamburg, Germany). VK2/E6E7 cells were grown in 35 mm poly-D-lysine–coated glass bottom dishes (MatTek Corp., Ashland, MA, USA) and maintained in medium containing penicillin/streptomycin (PEST). Cells were injected with a FemtoJet micromanipulation device (Eppendorf) for 0.5–1 s at 150 hPa. Following injection, cells were washed once in medium containing PEST and incubated at 37°C in 5% CO_2_ for 20–24 h. Cells were then fixed for 10 min in 3.7% PFA, permeabilized for 10 min in 20 mM Tris-HCl, pH 7.4, 50 mM NaCl, 3 mM MgCl_2_, 0.5% Triton X-100, and 300 mM sucrose, and blocked with 3% bovine serum albumin for 1 h at room temperature. A primary rabbit anti-53BP1 antibody (Nordic Biosite, Täby, Sweden) was used at 1∶300 dilution, and a secondary goat anti-rabbit IgG antibody conjugated to Alexa Fluor 633 nm (Molecular Probes, Life technologies, Paisley, UK) was used at 1∶500 dilution.

### Intracellular 3D rendering

VK2/E6E7 were grown in 35 mm poly-D-lysine–coated glass bottom dishes. Prior to infection, *N. gonorrhoeae* MS11 P+ were stained with DyLight 594 NHS Ester (Thermo Scientific, Waltham, MA, USA) for 7 min at 37°C in 5% CO_2_. Cells were then infected with stained *N. gonorrhoeae* for 10 h, stained with Hoechst 33342, (0.5 µg/ml) for 30 min at 37°C in 5% CO_2_, and washed twice with PBS. The dish was transferred to a live-cell incubator that was connected to an inverted microscope. Cells were maintained at 37°C in 5% CO_2_ throughout the microscopy analysis and monitored for additional 12 h to capture cells undergoing mitosis. Images of mitotic cells were taken through a 100x oil objective in 40–60 z-stacks with optimized optical thickness. Images were further deconvolved by an iteration algorithm and further processed by Inside 4D software Axiovision (Carl Zeiss, GmbH, Göttingen, Germany) and Photoshop CS5 (Adobe).

### Chromatin binding assay

Chromatin was extracted from VK2/E6E7 cells using the ChromaFlash Chromatin extraction kit (Epigentek, Farmingdale, NY, USA) according to manufacturer's instructions. Extracted chromatin was coated in a 96-well plate at a concentration equivalent to 1.25×10^5^ cells/well in PBS at 4°C. A subsequent ELISA was performed as follows: the plate was blocked in 3% non-fat dry milk for 1.5 h whereby PFA-fixed or viable MS11P+ suspended in PBS was added and incubated for 2 h at 37°C and 5% CO_2_. Non-adhered bacteria was washed away and chromatin-bound bacteria fixed by addition of 4% PFA for 15 min. Polyclonal antibodies rabbit anti- *N.gonorrhoeae* (MBS315050, MyBiosource, 1∶1000) was incubated for 45 min followed by 45 min with a goat anti rabbit IgG-HRP antibody (Bio-Rad, Hercules, CA, USA, 1.5000). TMB (Invitrogen, Camarillo, CA, USA) was added, and the HRP-TMB reaction stopped after 5 min with 1M HCL whereby absorbance was read at 450 nm.

### Time-lapse microscopy

Cell monolayers were grown to 30–40% confluence in 12-well poly-D-lysine–coated glass bottom dishes. *N. gonorrhoeae* MS11 P+ bacteria were added and incubated at 37°C with 5% CO_2_. After 2 h, unbound bacteria were washed away and dishes were transferred to a live-cell incubator at 37°C in 5% CO_2_, connected to an inverted fluorescence microscope. After an additional 1-h acclimatization period, randomly selected fields-of-view (containing 10–20 cells) were observed for 21 h with a 20 x objective. Differential interference contrast (DIC) images were captured every 10 min for each of the positions randomly chosen. Data were collected from 450 cells in three independent experiments. Images were further processed using ImageJ and Photoshop CS5 software.

### Cell synchronization and nuclear size assays

Epithelial cells were subcultured on poly-D-lysine-coated 0.17-mm coverslips (Gerhard Mentzel GmbH, Braunschweig, Germany). Cells were synchronized to the G1/S border using medium containing 2.5 mM thymidine for 28 h at 37°C and 5% CO_2_. Cells were subsequently released from the synchronization by substituting the thymidine-containing media with K-SFM for 12 h. Next, cells were again blocked with thymidine for 28 h at 37°C and 5% CO_2_. Cells were released from synchronization a second time, and infection was initiated by adding *N. gonorrhoeae* to the subconfluent cells. At 7 h post-infection, cells were fixed for 10 min in 3.7% paraformaldehyde (PFA). Coverslips were mounted in Vectashield containing 4′-6-Diamidino-2-phenylindole (DAPI, Vector laboratories, Burlingame, CA, USA). Fluorescent images of infected and control cells were captured with a CCD camera connected to an inverted fluorescence microscope (Cell Observer, Carl Zeiss, GmbH, Göttingen, Germany). Data were collected from 100 uninfected and 100 infected cells, ensuring that visible cell-adherent bacteria were present and that nuclei were in focus, in three independent experiments; that is, a total of 300 uninfected and 300 infected cells were analyzed. Nuclear areas were measured using the Axiovision software.

### Quantitative real time PCR analysis of target mRNAs in VK2/E6E7 cells

Following an initial screen using an RT^2^ Profiler PCR array (Qiagen) to study the expression of 96 cell cycle regulatory genes, a set of candidates was selected for further studies (data not shown). Bacteria were added to the subconfluent monolayers of VK2/E6E7 cells and allowed to adhere for 24 h. Unbound bacteria were washed away after the first 2 h of the incubation period. RNA from control cells and infected VK2/E6E7 cells was isolated with the RNeasy Mini kit according to the manufacturer's recommendations (Qiagen, Hilden, Germany). Total RNA (up to 1 µg) was reversed transcribed using oligo-dT primers and the Transcriptor First Strand cDNA Synthesis Kit (Roche, Basel, Switzerland), according to the manufacturer's recommendations. Primers used are listed in Table 2. QPCR amplification was performed using a Light Cycler 480 (Roche) and the SYBR Green I Master Kit (Roche), with 0.1 µM of each gene-specific primer and 5 µl of cDNA. Cycling conditions for PCR were as follows: initial denaturation for 5 min, followed by 30 cycles of denaturation at 94°C for 30 s, annealing at 55°C for 15 s, and extension at 71°C for 30 s. The ramp rate was 2.2–4.4°C/s. Relative expression levels of target gene mRNAs were calculated, normalized, and compared between control cells and infected cells. Expression levels of α-tubulin (*TUBA1A*) and glyceraldehyde 3-phosphate dehydrogenase (*GAPDH*) were used as internal standards to normalize expression levels of target genes.

**Table 2:** Primer sequences used for detection of human genes in VK2/E6E7 cells by qPCR.

Gene	Primer orientation	Primer sequence
*MAD1L1*	Forward	5′- AGG TTC TCT GCA GAT GCA GTA CCA -3′
	Reverse	5′- CTT GTG ACT AGC TCC ATC TGC AT -3′
*MAD2L1*	Forward	5′- AGG TCC TGG AAA GATGGC AGT TTG -3′
	Reverse	5′- TGT CAC CGT AGCTGT GAT CTG TCT -3′
*TUBA1A*	Forward	5′- ACA TCG ACC GCC TAA GAG TCG C-3′
	Reverse	5′- TGC ACT CAC GCA TGG TTG CTG-3′

### SDS-PAGE and western blotting

Infected and control cells were washed three times with PBS and harvested in 0.2% Igepal (Sigma-Aldrich Inc., St. Louis, MO, USA) containing 1× protease inhibitor cocktail (Roche) in PBS. Sample buffer (87% Glycerol, 1.5 M Tris-HCl pH 6.8, 10% 2-mercaptoethanol, and 0.1% SDS) was added to cell lysates, and samples were boiled for 10 min at 95°C prior to resolving on SDS-PAGE gels. SDS-PAGE was performed according to the manufacturer's protocol (Bio-Rad, Hercules, CA, USA). Protein gels were blotted onto Immobilon-FL PVDF membranes (Merck Millipore, Billerica, MA, USA). Membranes were blocked for 1 h in 5% non-fat dry milk in PBS (Bio-Rad). The following antibodies were used for immunoblotting: rabbit anti-MAD1L1 (Sigma-Aldrich Inc., St. Louis, MO, USA, 1∶100), mouse anti-MAD2L1 (17D10, Sigma-Aldrich, 1∶500), goat anti-rabbit IgG and goat anti-mouse IgG conjugated to IRdye800CW (Li-COR, Lincoln, Nebraska, USA) or IRdye680 (both 1∶10,000). Membranes were visualized and analyzed using an Odyssey IR scanner (Li-COR) at 700 or 800 nm. Immunoblot band intensities were quantified using ImageJ software. Polyclonal antibodies against α-tubulin (MBS316320, MyBioSource, 1∶1,000) or GAPDH (G-9545, Sigma-Aldrich, 1∶5,000) were used for normalizing total protein loaded in each well.

### Micronuclei assays

*N. gonorrhoeae* was added to a subconfluent monolayers of VK2/E6E7 cells and incubated for 24 h at 37°C in 5% CO_2_. Unbound bacteria were washed away and a final concentration of 5.6 µg/ml cytochalasin B (Sigma-Aldrich) was added to block cytokinesis. Cells were further incubated for 36 h at 37°C in 5% CO_2_. The cells were then washed in PBS and harvested with trypsin. Cells were pelleted by centrifugation for 15 min at 155 *x g* and supernatants were removed. Room temperature (RT) KCl (0.14 M; Merck, Darmstadt, Germany) was slowly added to cell pellets while vortexing, and cells were incubated with KCl at RT for 5 min. Cells were centrifuged for 10 min at 155 *x g*, and pellets were fixed by slowly adding fixative I (0.46% NaCl and 1% glacial acetic acid in methanol) while vortexing. Cells were incubated at RT for 5 min and centrifuged for 10 min at 155 *x g*. Supernatants were removed and fixative II (2.5% glacial acetic acid in methanol) was added. After a five minute RT incubation, cells were centrifuged for 10 min at 155*x g*, washed with the fixative II solution, spun down for 10 min at 155*x g*, suspended in fixative II, applied to glass slides (previously degreased with methanol), and left overnight to dry. Slides were stained with 5% Giemsa (Merck, Darmstadt, Germany) in PBS for 10 min. Excess Giemsa stain was washed away with double-distilled water, and the slides were left to dry overnight. A light microscope with a 40× objective was used for micronuclei scoring according to criteria set by M. Fenech [22]. A total of 1000 BNC from control or infected cells were scored for micronuclei in 3 independent experiments. The dispersion index (DI) was calculated by dividing the variance of the mean to aberrations per cell.

## Results

### Restriction endonucleases are upregulated during bacterial invasion

At least 10 restriction endonuclease genes are present in *N. gonorrhoeae* strain MS11 [21]. The expression of bacterial restriction endonucleases was measured by qPCR in *N. gonorrhoeae* MS11P+ upon infection. We chose four type II restriction endonucleases *NgoN1aIV, NgoMIV, NgoPI* and *NgoI* for further studies since type II restriction endonucleases were the most abundant among the genes found, one was commercially available, and they did not need additional supplements for digestion [23].

**Figure 1:**
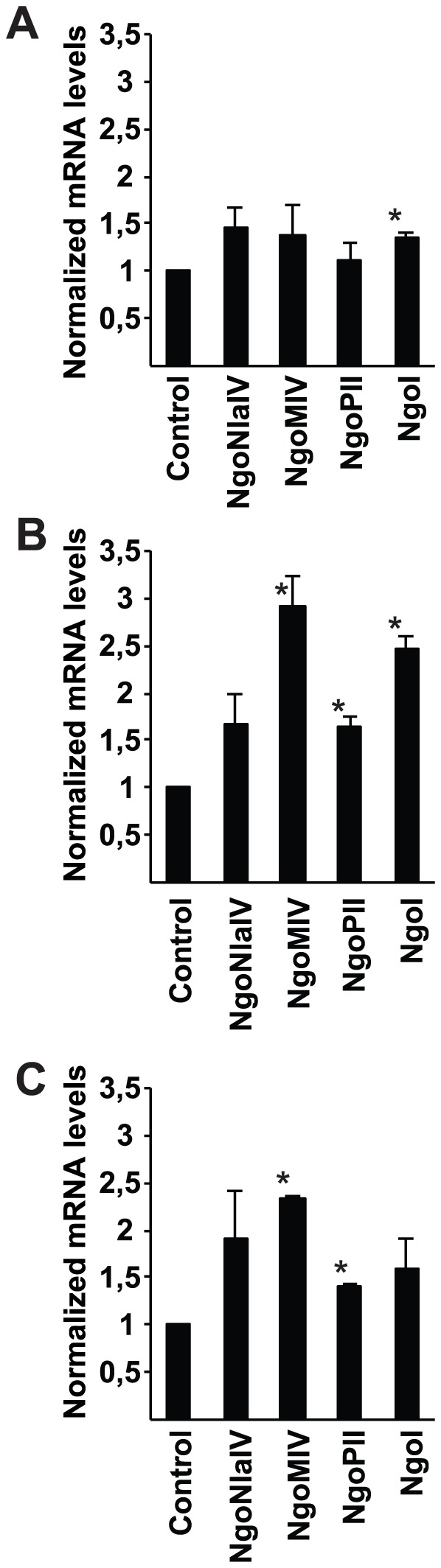
Bacterial restriction endonucleases are upregulated upon adhesion and invasion. Cells were infected with *N. gonorrhoeae* strain MS11P+ for 2, 7, and 24 h. Bacteria alone (without cells) were used as a control. qPCR was conducted on bacterial DNA, and *Ef-Tu1* was used as internal standard. Average normalized mRNA levels of *NgoNlaIV*, *NgoMIV*, *NgoPII*, and *NgoI* following A. 2 h. B. 7 h, and C. 24 h of infection in 2 independent experiments are shown. Data were analyzed using a paired 2-tailed Student's *t*-test. Error bars represent S.E.M. (*p<0.05).

Gonococci were allowed to infect VK2/E6E7 cells for 2, 7, and 24 h. As a control, bacteria were incubated without cells in cell culture media, under the same conditions. RNA was stabilized, isolated, and qPCR was performed. After a 2 h infection period, the expression of *NgoI* was upregulated 1.4-fold (Fig. 1A). After 7 h, three out of the four restriction endonucleases, that is, *NgoMIV* (2.9-fold), *NgoPII* (1.7-fold), and *NgoI* (2.5-fold) were upregulated (Fig. 1B). This effect declined after 24 h, when *NgoMIV* and *NgoPII* were still upregulated (2.4-fold and 1.4-fold, respectively), compared to the control (Fig. 1C). The qPCR values were normalized against the Elongation factor thermo unstable 1 gene (*Ef-Tu1*), and data were analyzed using a paired 2-tailed Student's *t*-test. The same trend could be seen using *pgm* as an internal standard (data not shown). Taken together, these data show that bacterial infection of host cells triggers bacterial restriction endonuclease expression, with highest upregulation observed at 7 h post-infection, a time-point that correlates with high bacterial invasion (Fig. S1).

### Restriction endonucleases in bacterial lysate fragments DNA in vitro

**Figure 2:**
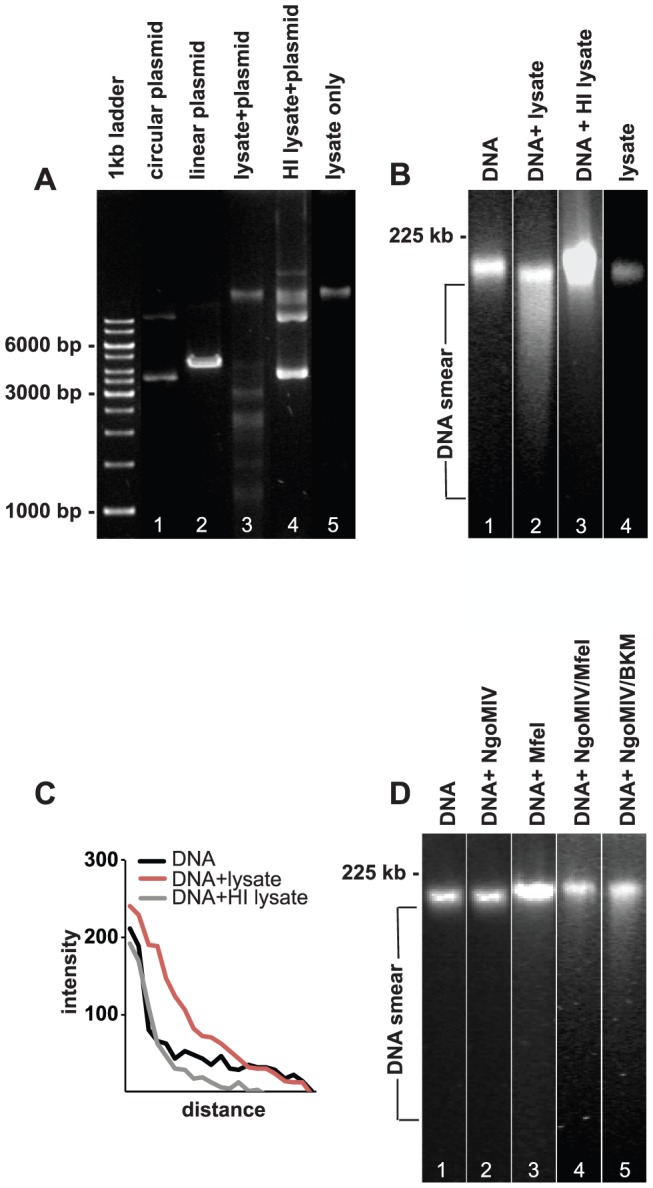
Lysates of *N. gonorrhoeae* fragments pECFP-N1 and damage DNA from VK2/E6E7 cells. A. DNA agarose gel showing the digestion of pECFP-N1 plasmid by HindIII (positive control, lane 2), MS11 P+ lysate (lane 3), and MS11 P+ HI lysate (lane 5). Lane 5 shows bacterial MS11 P+ lysate without pECFP-N1 and lane 1 shows uncut circular pECFP-N1. B. PFGE analysis of purified VK2/E6E7 genomic DNA treated for 24 h with: lane 1: PBS (negative control), lane 2: MS11 P+ lysate, lane 3: MS11 P+ HI lysate. Lane 4 shows bacterial MS11 P+ lysate without VK2/E6E7 genomic DNA. C. Graph showing quantification of DNA smears (measured directly underneath and below the band). Shown are smear pixel intensities of cellular DNA alone and cellular DNA exposed to bacterial lysates and HI bacterial lysates. D. PFGE showing genomic DNA subjected to commercial restriction enzymes for 24 h. Lane 1: DNA incubated with CutSmart reaction buffer (negative control). Lane 2: DNA incubated with NgoMIV. Lane 3: DNA incubated with MfeI, Lane 4: DNA incubated with NgoMIV and MfeI Lane 5: DNA incubated with NgoMIV and BamHI/KpnI/MfeI (BKM).

Intracellular gonococci can be found in the cytoplasm of infected cells during interphase. However, intracellular bacteria can undergo autolysis in the host target cell. To see if the triggered restriction endonuclease production by intracellular bacteria can damage host cell DNA, we first lysed a bacterial suspension by repeated freeze-thaw cycles. To confirm that the lysates contain active restriction endonucleases, the MS11 P+ lysate was mixed with a common plasmid pECFP-N1 for 1 h at 37°C. Indeed, the plasmid was fragmented into several smaller fragments, clearly showing that the lysate contained several active restriction endonucleases enable to cut double stranded DNA (Fig 2A). As negative control, we used heat-inactivated (HI) MS11 P+ lysate, leaving pECFP-N1 in a circular form. The specific fragmentation of the plasmid DNA at clear recognition sites confirms that active endonucleases were responsible for the DNA cutting, thereby disqualifying other possible DNA degrading enzymes such as exonucleases.

Then, genomic DNA was extracted from VK2/E6E7 cells and treated with solutions containing bacterial lysates or control medium for 24 h at 37°C and were immediately analyzed by pulsed-field gel electrophoresis (PFGE). The only commercially available MS11 restriction enzyme NgoMIV was used; however, this enzyme is sensitive to methylated DNA. Therefore, we also used BamHI, KpnI, and MfeI as positive controls for successful fragmentation of genomic DNA.

MS11P+ lysates were able to fragment/smear human genomic DNA, as visualized by smear in PFGE (Fig. 2B). DNA fragmentation was abolished when treated with HI bacterial lysate. Quantification of smears clearly showed that human genomic DNA treated with gonococcal lysates display the strongest pixel intensity (highest smearing), when compared to smearing observed following incubation with HI lysates (Fig. 2C). Neither the commercial restriction enzyme MfeI nor NgoMIV was able to digest genomic DNA alone (Fig. 2D). A moderate smear was detected when both NgoMIV and MfeI were added together. An even stronger smear was detected when the four restriction enzymes NgoMIV, BamHI, KpnI and MfeI were used together (Fig. 2D).

Thus, the bacterial lysates contain sufficient amounts of restriction endonucleases to fragment both plasmids as well as purified host cell DNA. Most likely, a synergistic effect of the restriction endonucleases in the bacterial lysates contributes to the strong smearing.

### Bacterial lysates microinjected into cell cytoplasm transvers into the nucleus and cause DNA DSBs

**Figure 3:**
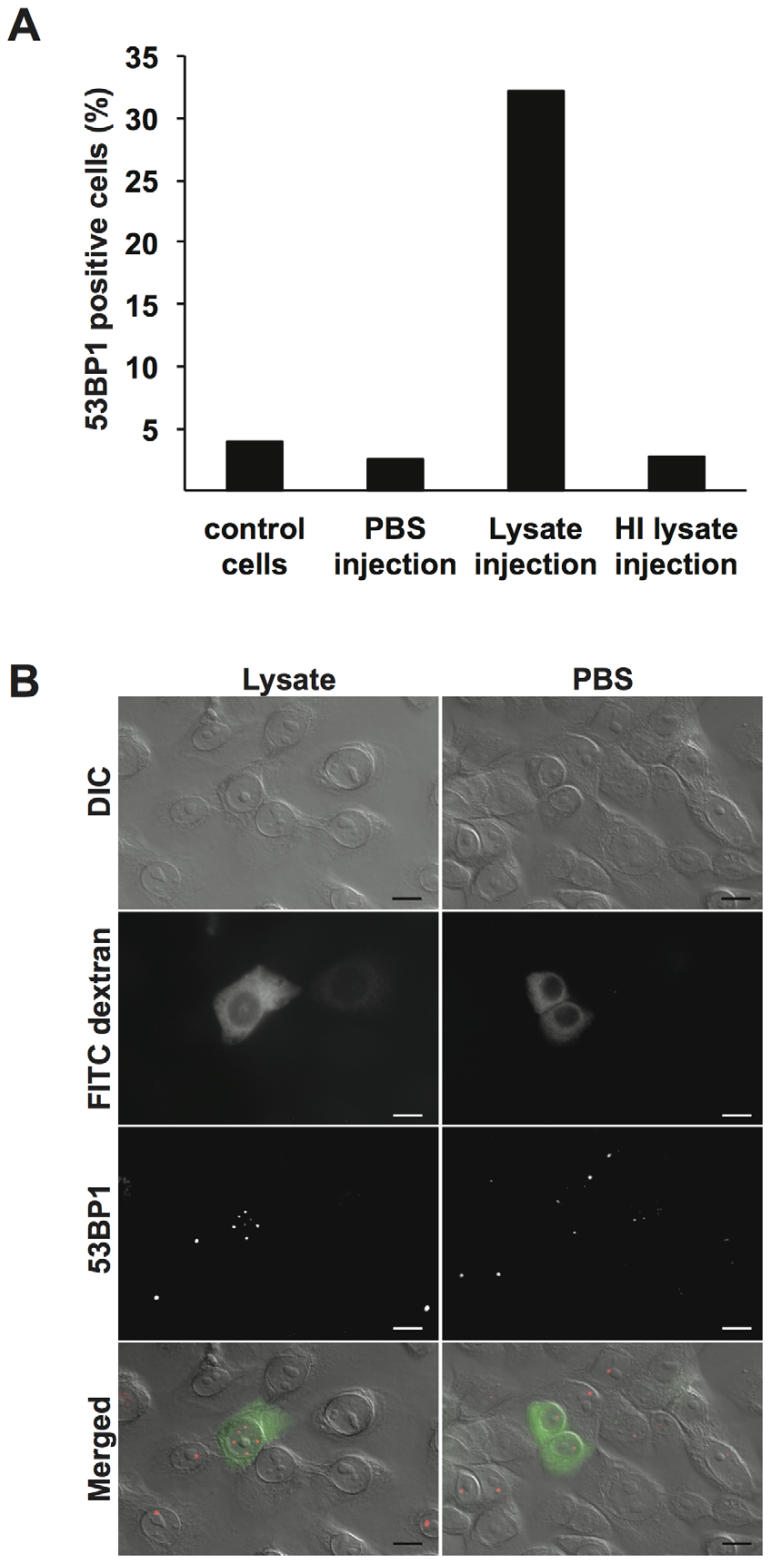
Microinjection of bacterial lysates in the cytoplasm of VK2/E6E7 cells causes DSBs. A. Interphase VK2/E6E7 cells were subjected to cytoplasmic microinjection of bacterial MS11 P+ lysate, MS11 P+ HI lysate, or PBS. Cells were incubated for 20–24 h and then stained for DSBs with 53BP1 antibodies. The graph shows the average number of 53BP1 positive cells counted in two independent experiments under each condition. Control cells are non-injected cells. B. Images showing DIC and fluorescent images of representative cells microinjected with MS11 P+ lysate (left) or PBS (right). FITC-dextran (green) was co-injected into the cytoplasm to identify microinjected cells. Scale bar represents 10 µm.

Bacterial lysates were centrifuged to remove precipitates and was then microinjected into the cytoplasm of VK2/E6E7 cells in interphase. Cells were then incubated at 37°C in 5% CO_2_. As negative controls, PBS or HI lysates were microinjected in an identical manner. FITC-coupled dextran was co-injected to identify microinjected cells. After 20–24 h of incubation, cells were fixed and stained with antibodies against p53 binding protein 1 (53BP1). The 53BP1 specifically localizes to DNA DSBs. No fragmented DNA or apoptotic bodies were detectable during the assay. Occasionally, incubations were extended to over 30 h to ensure that no apoptosis was present. The number of 53BP1-positive foci was counted in both microinjected (FITC-dextran positive cells) and control cells (non-injected). Any cell containing 4 foci or more was deemed positive. Out of 43 and 53 injected cells studied in two independent experiments, an average of 32% were positive for 53BP1 after microinjection with bacterial lysates (Fig. 3A). Cells injected with PBS (n = 40 and 41 cells counted in two independent experiments) and control cells (n = 321 and 136 cells counted) had an average of 4% and 2.4% 53BP1 positive cells, respectively (Fig. 3A). Injection of HI lysates resulted in 2.6% 53BP1 positive cells (n = 38 cells counted). Representative microscopic images of a 53BP1 positive cell microinjected with bacterial lysates and 53BP1-negative cells injected with PBS are shown in figure 3B. These data clearly shows that the restriction endonucleases in the bacterial lysates are able to cross the nuclear membrane and target the host cell DNA and cause DSBs.

### Subcellular location of intracellular bacteria in mitosis

**Figure 4:**
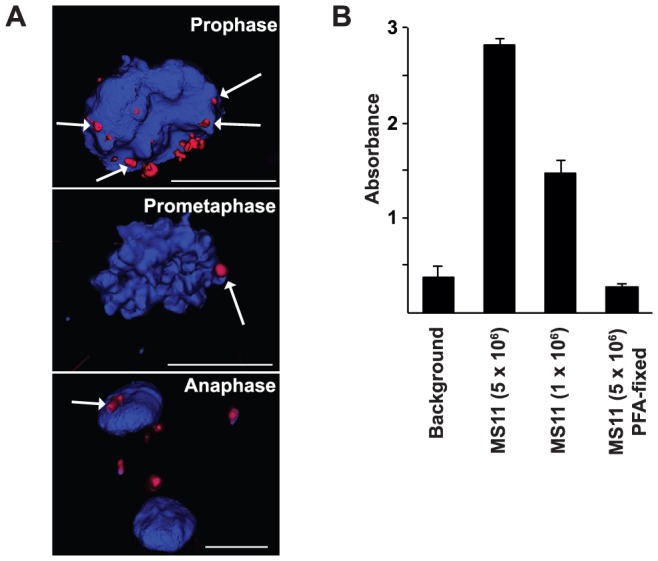
Subcellular location of intracellular bacteria in mitosis. A. VK2/E6E7 cells were infected with DyLight 594 NHS Ester-stained *N. gonorrhoeae*. Cells were maintained in a live-cell incubator connected to an inverted fluorescence microscope. Cellular DNA was stained with Hoechst 33342 and is shown in blue. Images of live mitotic cells were taken through a 100x oil objective in 40–60 z-stacks. Images were further deconvolved and processed by Inside 4D software. Bacteria (red) present during prophase (upper), prometaphase (mid) and anaphase (lower) are shown. Bacteria that are in close proximity to the chromatin are marked with arrows. The scale bar represents 10 µm. B. Viable bacteria associate with chromatin in ELISA. Chromatin, extracted from 1.25×10^5^ cells was coated in each well. Viable (5×10^6^ CFU/well or 1×10^6^ CFU/well) or PFA-fixed (5×10^6^ CFU/well) MS11P+ was allowed to interact with the chromatin for 2 h at 37°C and 5% CO_2_. Rabbit anti- *N. gonorrhoeae* antibodies followed by goat anti rabbit IgG-HRP antibodies were used to detect the bacteria bound to the chromatin. Absorbance was read at 450 nm. Background was measured in wells containing all agents except chromatin. Shown is a graph of one assay in technical triplicate and standard deviations.

During infection, the bacteria invade the host cell and reside in the cytoplasm. While some bacteria may undergo autolysis there are still several viable bacteria present. Once the cell enters mitosis, the nuclear envelope breaks down, allowing intracellular bacteria access to condensed chromatin. Hence, we investigated where bacteria are localized in mitotic cells. VK2/E6E7 cells were infected with *N. gonorrhoeae* in a live-cell incubator connected to an inverted microscope. Images of mitotic cells were taken through a 100x oil objective in 40–60 z-stacks with optimized optical thickness. Interestingly, we could see gonococci in the vicinity of host cell chromatin during prophase, prometaphase, and anaphase (Fig. 4A). Bacteria could also be detected at a distance of a few micrometers away from chromatin, suggesting that bacteria can be found close to the human genome during mitosis. To further confirm the bacteria-chromatin association, bacteria were allowed to bind purified chromatin from VK2/E6E7 cells in an ELISA. Indeed, viable, but not PFA-fixed, MS11 bound extracted chromatin showing that the bacterial affinity for human chromatin is dependent on live bacteria (Fig. 4B). Thus, intracellular bacteria may possess two modes of affecting the host cell genome; via restriction endonucleases to generate DSBs and interfering with the chromatin.

### The bacterial infection impairs mitotic progression by distorting MAD1/MAD2 complexes

**Figure 5:**
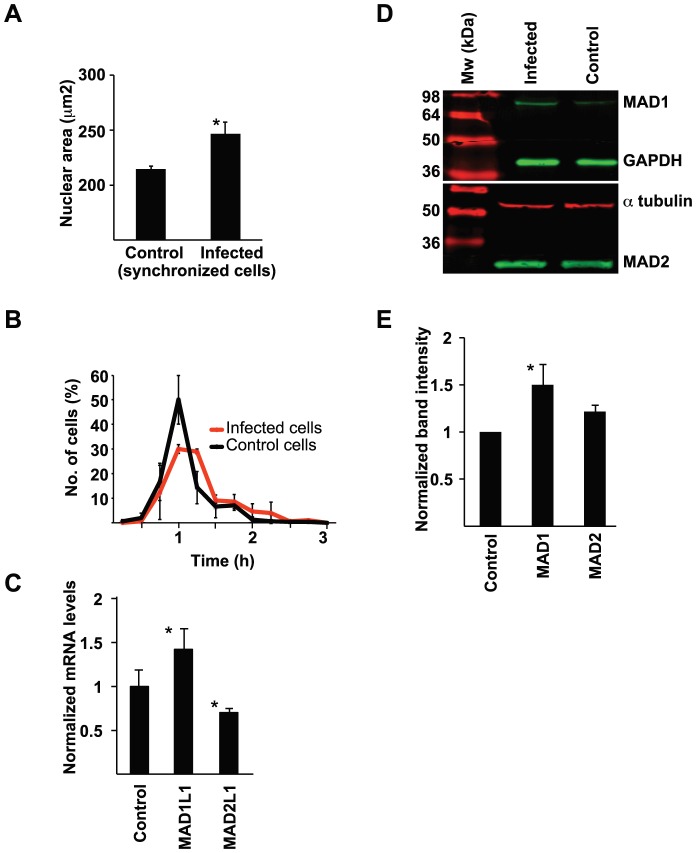
Gonococcal infection causes durations of mitosis, nuclear swelling, and targets regulatory mitotic genes and proteins. A. VK2/E6E7 cells were synchronized and bacteria were allowed to adhere and invade cells for 24 h. Cells were then fixed and stained with DAPI. The nuclear area of 300 infected and 300 uninfected cells were measured. Data were analyzed using a paired 2-tailed Student's *t*-test. The average nuclear areas observed in 3 independent experiments are shown (*p<0.05). Error bars represent S.E.M. B. Cells were infected with *N. gonorrhoeae* and observed for 24 h by live-cell microscopy. DIC images were captured every 15 min at randomly selected positions, and times spent transitioning from prophase to cytokinesis were measured for 150 mitotic cells. The graph shows mean ± standard deviation values for the time required for progression from prophase to cytokinesis, measured in 3 independent experiments. C. VK2/E6E7 cells were infected with *N. gonorrhoeae* for 24 h. RNA from control cells and infected cells was isolated and qPCR was performed. Average mRNA levels of *MAD1L1* and *MAD2L1* from 3 independent experiments are shown (*p<0.05). Error bars represent S.E.M. Values were normalized against *TUBA1A* expression and analyzed using a paired 2-tailed Student's *t*-test. D. Protein expression in infected cells and control cells was analyzed in western blots using antibodies against of MAD1 and MAD2. GAPDH or α-tubulin was used as loading controls. E. Western blot band intensities were quantified and analyzed with a paired 2-tailed Student's *t*-test, using GAPDH or α-tubulin expression as controls. Mean expression levels of each protein observed in 3 independent western blots are shown (*p<0.05). Error bars represent S.E.M.

Due to the DSBs caused by the restriction endonucleases we further analyzed what consequences the infection has on nuclear morphology and mitosis regulation. Cells were infected with *N. gonorrhoeae* and analyzed for mitosis regulatory genes and proteins or monitored by live-cell microscopy. First, nuclear areas were measured in 300 randomly selected control cells or infected cells. Infected cells displayed average nuclear areas of 246 µm^2^, compared to control cells with average nuclear areas of 214 µm^2^ (Fig. 5A). This result shows that the gonococcal infection causes nuclear swelling of the host target cell. Second, differential interference contrast (DIC) images were captured every 10 min during live-cell imaging and durations between prophase to cytokinesis were measured. The majority of uninfected control cells spent 1 h in mitosis; none of these cells spent >2 h in mitosis. In contrast, the majority of infected cells spent at least 1 h and 15 min mitosis, and some took as long as 2 h and 45 min (Fig. 5B). Thus, cells undergo prolonged mitosis during bacterial infection.

Further, the gonococcal infection upregulated *MAD1L1* mitotic arrest deficient-like 1 (encoding the MAD1 protein) by 1.4-fold and downregulated *MAD2L1* mitotic arrest deficient-like 1 (encoding the MAD2 protein) by 0.7-fold (Fig. 5C). The qPCR values were normalized against *TUBA1A* mRNA expression, and data from 3 independent experiments were analyzed using a paired 2-tailed Student's *t*-test. The same trend was seen using *GAPDH* as internal standard (data not shown). Protein levels of MAD proteins were further analyzed by SDS-PAGE and immunoblotting using antibodies against MAD1 and MAD2. MAD1 was increased by 1.5-fold, compared to the GAPDH internal standard (Fig. 5D and E). In conclusion, infected cells overexpress MAD1 and thereby distort the important ratio between the MAD1/MAD2 complex that lead to altered mitosis.

Taken together, the bacterial infection distorts the MAD1/MAD2 ratio important for metaphase regulation and timing. Infected cells suffer from prolonged mitotic progression and nuclear swelling.

### The bacterial infection causes micronuclei formation

**Figure 6:**
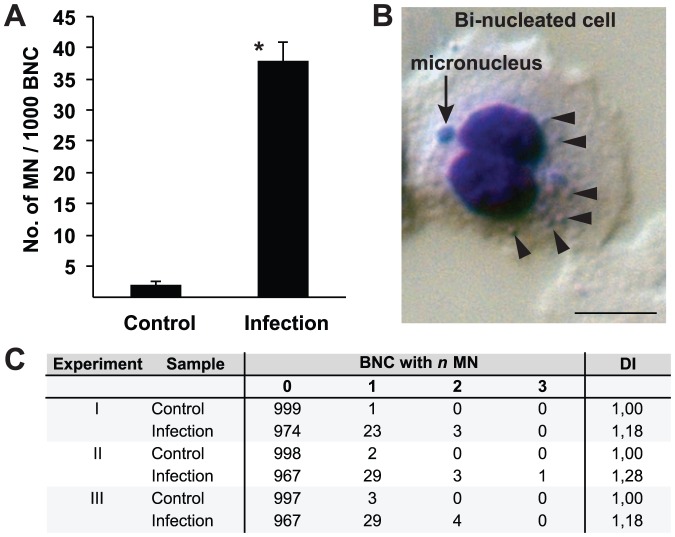
Bacterial infection induces micronuclei formation in VK2/E6E7 cells. VK2/E6E7 cells were infected with *N. gonorrhoeae* for 24 h. Cytokinesis was blocked with cytochalasin B for 36 h and BNC from infected and control cells were analyzed for micronuclei formation. A. Average numbers of observed micronuclei/1000 BNC ± standard deviation from 3 independent experiments are shown (*p<0.05). B. DIC image showing a representative Giemsa-stained BNC (blue), containing one micronucleus (arrow) and several intracellular bacteria (arrowheads), captured using a 63× objective. Scale bar represents 10 µm. C. Frequencies of micronuclei formation and DI observed in 3 independent experiments where 1000 BNC were scored in each experiment are shown.

Micronuclei are small, extra-nuclear bodies formed during mitosis from parts of chromosomes either as consequences of DNA double strand breaks or of damage to nuclear division apparatus [24]. We performed micronuclei assays with infected and control VK2/E6E7 cells. Cells were infected with gonococci for 24 h. Cytochalasin B was added to block cytokinesis and the cells were incubated for another 36 h to generate bi-nucleated cells (BNC). Infected cells showed an average of 38 micronuclei per 1000 BNC, whereas uninfected control cells displayed an average of 2 micronuclei in 1000 BNC (Fig. 6A). A representative DIC image of a BNC containing two micronuclei and several intracellular bacteria present is shown in figure 6B. In the majority of micronuclei-positive infected BNC, one micronucleus was present, although some infected BNC had up to three micronuclei present, indicative of more severe damage to nuclei (Fig. 6C). Notably, infected cells showed a reduced frequency of BNC after 36 h of cytochalasin B treatment, due to the deceleration of the cell cycle and prolonged mitosis times. Over 50% of control cells had completed cytokinesis and were bi-nucleated, while only 8% of the infected cells were. These results clearly show that infected cells undergo mitosis at slower rates (Fig. S2).

Further, infection-induced compromised chromosome segregation could be seen in forms of lagging chromosomes in late anaphase cells that were infected with DyLight 594 NHS ester-stained gonococci (Fig. S3). The progression through anaphase was followed from mid- to late anaphase in infected cells and the timespans for lagging chromosomes were in some cases protracted up to several hours (Fig. S3).

Taken together, the DSB caused by the restriction endonucleases and possible interaction of the bacteria on the chromatin cause micronuclei formation epithelial cells.

## Discussion

**Figure 7:**
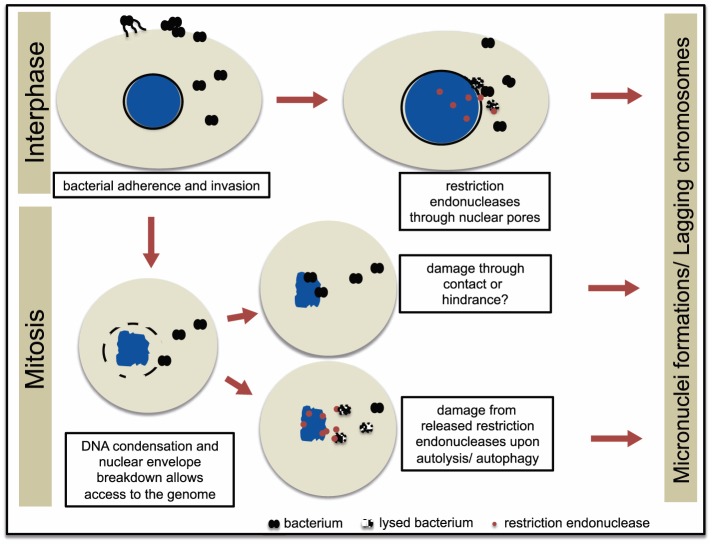
Proposed model of *N. gonorrhoeae*-induced DNA damage in epithelial cells. *N. gonorrhoeae* adheres to and invades epithelial cells. Restriction endonucleases are upregulated and released from intact bacterium or through bacterial autolysis during invasion and access the nucleus through nuclear pores during interphase. During early mitosis when the nuclear envelope is broken down, *N. gonorrhoeae* may interact with the condensed chromatin, which affects mitosis. Bacterial restriction endonucleases released from the bacterium also access the chromatin in mitosis. The result of each scenario is micronuclei formation, ultimately causing damaged DNA in the host cell.

Commercial restriction enzymes have been widely used in cancer research where researchers have shown that restriction endonucleases can pass through nuclear pores to gain direct access to the DNA, capable of digesting methylated human DNA in both interphase and mitosis [25] and also cause DSBs leading to chromosomal instability [26], [27]. The use of commercial restriction enzymes (primarily AluI and PvuII) has been used for a long time in research in DNA damage and radiation biology. In these articles they clearly show that the restriction enzymes (introduced in the cytoplasm by electroporation) enters the nucleus and are solid responsible for double strand DNA breaks and chromosomal aberrations to CHO cells [28]–[30]. Hence, the presence of REs in the nucleus after microinjection of the bacterial lysate is reasonable to assume. Naturally, many bacteria produce abundant amounts of restriction endonucleases to gain an efficient restriction barrier against foreign DNA. Although the majority of gonococcal restriction endonucleases were identified several decades ago, the endogenous role of these conserved genes in virulence and pathogenesis are elusive. Gonococci are remarkably autolytic due to the expression of lytic transglycosylases [31]–[33], and gonococcal restriction endonucleases can be released into the host cell cytoplasm when bacteria lyse inside the host cell. We performed qPCR analysis on four restriction endonucleases in the *N. gonorrhoeae* transcriptome. We observed upregulation of NgoMIV, NgoPII and NgoI upon bacterial invasion. Interestingly, no upregulation was seen for NgoNlaIV, a restriction endonuclease present in *N. gonorrhoeae* MS11 but acquired from commensal *Neisseria lactamica*. Most likely, a synergistic effect is present in the bacterial lysate, with more than four restriction endonucleases active capable of digesting purified genomic DNA of host cells as detected by PFGE. The data was supported by microinjecting bacterial lysates into the cellular cytoplasm of living cells. The restriction endonucleases in the lysate were able to cross the nuclear membrane and caused DSBs (Fig. 7). It is likely that the transversal of restriction endonucleases into the nucleus increased the permeability and chromatin decondensation in stressed nuclei [34] as reported for bacterial infections [35]. However, the nuclear swelling upon gonococcal infection may also be due to the infection-induced carcinogenic effect. Measuring nuclear size is a well-established method to elucidate potential carcinogens. Host cell nuclear swelling is a rapid event occurring within hours upon addition of carcinogenic agents. We find this very intriguing since clinical reports have correlated gonococcal infections with urogenital cancers.

A growing number of studies show that intracellular bacteria target the nucleus either by secreting nucleomodulins or directly invading the nucleus [15]. We show that *N. gonorrhoeae* has the potential to exert nucleomodulatory effects by being in close proximity to cellular chromatin during different mitotic phases. During a 24 h gonococcal infection, an infected host cell most likely undergoes mitosis once, allowing the intracellular bacteria access to the genome. It is possible that the interaction is the appropriate location to also efficiently access the chromatin to reprogram the expression of host genes such as transcription factors and histone modifications, as previously shown during *Shigella flexneri* infections [36] and *Chlamydia trachomatis* infections [37].

We show that the infection changes the ratio between mitotic checkpoint proteins MAD1 and MAD2. Heterodimers of MAD1 and MAD2 proteins on the unattached kinetochores produce a “wait anaphase” signal to APC/C until proper alignment between microtubules and kinetochores of sister chromatid pairs occurs [38], [39]. A changed expression ratio results in a dysfunctional mitotic checkpoint complex (MCC) and chromosomal instability (CIN) as an ultimate outcome [40], [41]. We propose that the dysregulation of MAD1/MAD2 complex is a major cause for delayed mitotic progression in infected cells. *N. gonorrhoeae* is not the only bacterium that distorts the MCC. IpaB, a *Shigella* effector, targets MAD2B (encoded by the *MAD2L2* gene) and delays mitotic progression [42].

As a consequence of the bacterial infection, vaginal epithelial cells have approximately 19 times more micronuclei compared to control cells. Micronuclei are biomarkers for CIN that arise during anaphase from lagging acentric chromosomes or chromatid fragments caused by unrepaired DNA breaks and/or changes in kinetochore proteins, dysfunctional spindle, and defective anaphase checkpoint genes [43]. Our results obtained here indicate that restriction endonucleases do induce the DNA damage. Hence, it is reasonable to assume that micronuclei also are a consequence of this damage (Fig. 7).

Taken together, we propose a model that gonococcal infection cause damage to host cell DNA via two mechanisms; the presence of the bacteria close to the chromatin upon mitosis while the nuclear envelope is broken down and by restriction endonucleases that are released from the bacterium through out the whole cell cycle. This results in both DSBs and aberrant metaphase alignment due to altered expression of MAD proteins in the MCC complex. Considering the fact that *N. gonorrhoeae* also possesses type-I, and type-III restriction modification systems as well as new uncategorized putative DNA restriction endonucleases, the magnitude of their actions during infection is far from unraveled.

## Supporting Information

Figure S1 ***N. gonorrhoeae*** **adheres to and invades VK2/E6E7 cells.** Bacteria were allowed to adhere to and invade VK2/E6E7 cells for 2, 4, 6, or 24 h. Infected cells were treated with 1% saponin (Sigma), serially diluted, and spread onto GCB plates. A. Shown is mean CFUs/well of adherent bacteria ± standard deviation from 3 independent experiments. B. For invasion assays, Gentamycin (Sigma, 200 µg/ml) was added for the last hour of incubation. Shown is mean CFUs/well of intracellular bacteria ± standard deviation from 3 independent experiments.
(EPS)

Figure S2 **Gonococcal infection results in fewer BNC in VK2/E6E7 cells.** A. VK2/E6E7 cells were infected with *N. gonorrhoeae* for 24 h, treated with cytochalasin B for 36 h to block cytokinesis, and then analyzed for micronuclei. The percentage of BNC in 1000 uninfected and 1000 infected cells for each experiment are shown. B. The graph shows the average number of BNC generated in 3 independent experiments. Data were analyzed using a paired 2-tailed Student's *t*-test. Error bars represent standard deviation (*p<0.05).
(EPS)

Figure S3 **Lagging chromosome.** An image of a representative infected cell in anaphase was captured with a 100x oil objective in 40–60 z-stacks, deconvolved, and processed by Inside 4D software. One infected cell with a lagging chromosome in early anaphase (left) and then in late anaphase (right) is shown. DNA was stained with Hoechst's 33342 (blue) and bacteria were stained with DyLight 594 NHS Ester (red). Scale bar represents 1 µm.
(EPS)

Material and Methods S1 **Adherence and invasion assays.** Piliated bacteria were collected from GCB agar plates and resuspended in PBS. Optical density were measured at 600 nm to calculate the number of bacteria/ml. Bacteria were added to subconfluent monolayers of VK2/E6E7 cells and grown 37°C in 5% CO_2_ for 2, 4, 6, or 24 hours. Subsequently, cell monolayers were extensively washed with PBS until no unbound bacteria were visible by light microscopy. Infected cells were treated with 1% saponin (Sigma), serially diluted, and spread onto GCB plates. Plates were incubated at 37°C in 5% CO_2_ and the number of colony forming units (CFU) was counted after 2 days. For invasion assays, Gentamycin (Sigma, 200 µg/ml) was added for the last hour of incubation.
(DOCX)
