# Supplementary material for: Restriction Endonucleases from Invasive Neisseria gonorrhoeae Cause Double-Strand Breaks and Distort Mitosis in Epithelial Cells during Infection
Source: PLoS One. 2014 Dec 2;9(12):e114208. doi: 10.1371/journal.pone.0114208 (PMC4252111; doi:10.1371/journal.pone.0114208)
Supplement: Material and Methods S1 — Adherence and invasion assays. Piliated bacteria were collected from GCB agar plates and resuspended in PBS. Optical density were measured at 600 nm to calculate the number of bacteria/ml. Bacteria were added to subconfluent monolayers of VK2/E6E7 cells and grown 37°C in 5% CO2 for 2, 4, 6, or 24 hours. Subsequently, cell monolayers were extensively washed with PBS until no unbound bacteria were visible by light microscopy. Infected cells were treated with 1% saponin (Sigma), serially diluted, and spread onto GCB plates. Plates were incubated at 37°C in 5% CO2 and the number of colony forming units (CFU) was counted after 2 days. For invasion assays, Gentamycin (Sigma, 200 µg/ml) was added for the last hour of incubation. (DOCX) [file pone.0114208.s004.docx]

**Supplementary materials and methods**

**Adherence and invasion assays**

Piliated bacteria were collected from GCB agar plates and resuspended in PBS. Optical density were measured at 600 nm to calculate the number of bacteria/ml. Bacteria were added to subconfluent monolayers of VK2/E6E7 cells and grown 37°C in 5% CO_2_ for 2, 4, 6, or 24 hours. Subsequently, cell monolayers were extensively washed with PBS until no unbound bacteria were visible by light microscopy. Infected cells were treated with 1% saponin (Sigma), serially diluted, and spread onto GCB plates. Plates were incubated at 37°C in 5% CO_2_ and the number of colony forming units (CFU) was counted after 2 days. For invasion assays, Gentamycin (Sigma, 200µg/ml) was added for the last hour of incubation.
